# Interleukin 28B Gene Polymorphism and Association with Chronic Hepatitis C Therapy Results in Latvia

**DOI:** 10.1155/2012/324090

**Published:** 2012-04-24

**Authors:** Ieva Tolmane, Baiba Rozentale, Jazeps Keiss, Ludmila Ivancenko, Nadezda Subnikova, Zaiga Reinholde, Ieva Kozlovska, Nina Sumlaninova, Sniedze Laivacuma, Raimonds Simanis

**Affiliations:** ^1^Department of Hepatology, Infectology Center of Latvia, Riga, Linezera Street 3, 1006 Riga, Latvia; ^2^Division of Doctoral Studies, Riga Stradins University, Dzirciema Street 16, 1007 Riga, Latvia; ^3^Department of Infectology and Dermatology, Riga Stradins University, Dzirciema Street 16, 1007 Riga, Latvia; ^4^Faculty of Continuing Education, Riga Stradins University, Dzirciema Street 16, 1007 Riga, Latvia

## Abstract

*Introduction*. With the standard treatment of chronic hepatitis C, sustained virological response (SVR) can be achieved only in half of all patients. Interleukin-28B appears to be involved in the control of HCV infection, and the genetic polymorphism of the encoding IL-28B gene may determine the efficacy of clearance of HCV. The aim of this paper was to detect IL-28B gene polymorphism in Latvia and to analyze therapy results. This is the first study on IL-28B gene polymorphism in Latvia. *Material and Methods*. There were 159 chronic viral hepatitis C patients included in the study. In order to detect IL-28B gene polymorphism, we used molecular biology techniques and methods: classical DNA separation, amplification by PCR, and standard sequencing. Genotype was defined as CC, CT, TC, or TT type. 142 patients were treated with the standard of care treatment. Results were analyzed according to IL-28B polymorphism. *Results*. There were 53 patients (33%) with CC genotype, 84 patients (53%) with CT/TC genotype, and 22 patients (14%) with TT genotype. 34 patients (74%) in CC genotype subgroup achieved SVR versus 50 patients (52%) in non-CC subgroups. In patients with genotype 1, SVR was achieved in 16 patients (84%) in CC subgroup versus 30 patients (47.6%) in non-CC subgroups, *P* = 0.007. *Conclusions*. The most common genotype of IL28B in Latvia is CT/TC, with an incidence of 53%. Patients with CC genotype achieved SVR more often than CT or TT subgroups. IL28B gene polymorphism therefore is a strong predictor of treatment result.

## 1. Introduction

Chronic viral hepatitis C is one of the most serious chronic infections affecting 170 million people worldwide. In Europe more than 9 million people are infected with this virus [1]. The prevalence of hepatitis C virus (HCV) infection is also relatively high in Latvia. Antibodies are found in 2.4% of the population, with HCV-RNA prevalence of 1.7% of general population [2]. The outcome of HCV infection varies from spontaneous viral clearance, symptom free-HCV carrier state to chronic hepatitis, cirrhosis, and hepatocellular carcinoma. Some individuals have rapidly progressive liver disease while others remain in good health for many years. The reasons for different outcomes are poorly understood. There are a lot of factors such as viral kinetics, immune mechanisms, environmental factors, and patient-related factors which may be responsible for the various HCV-related outcomes.

Hepatitis C virus spreads mainly via blood. Risk factors for infection include intravenous and intranasal drug use, nonsterile tattooing, manicure and pedicure procedures, as well as virus transmission vertically from mother to child, and during sexual intercourse. Dialysis patients appear to be at increased risk of infection as do alcoholics [1, 3]. In developing and transitional economy countries, the nosocomial transmission of new hepatitis C virus (HCV) infections is a major problem due to reuse of contaminated or inadequately sterilized syringes and needles used in medical, paramedical, and dental procedures; an estimated 2.3–4.7 million new infections occur each year [1].

Current standard of care treatment involves combination therapy with hopes of effective virus eradication resulting in a sustained virological response (SVR) and thereby stopping the progression of disease. With the combination of pegylated alpha interferon and ribavirin, a sustained virological response can be achieved in only 54 to 63% of patients [4].

Cytokines have a role in the mechanisms of host defense against infectious agents. They induce inflammatory responses that can lead to tissue injury and serve as antiviral effectors as well. Cytokine synthesis is regulated by genetic mechanisms resulting in differences between individuals in their ability to produce cytokines. This individual variability may be due to single-nucleotide polymorphisms within the coding regions of cytokine genes. Cytokine genes are polymorphic, and some of these variants modify the production of the specific cytokines thereby affecting the host immune response. In HCV infection, secretion of inappropriate amounts of cytokines may be associated with developments of chronic disease or resistance to interferon (IFN) treatment. Interleukin-28B (IL-28B), named also as interferon *λ*-3, has been shown to be involved in the control of HCV infection, and the genetic polymorphism of the encoding IL-28B gene may determine the clearance of HCV. The IL-28B gene on human chromosome 19q was discovered in 2003, using the genomic screening process in which the entire human genome was scanned for putative genes [5, 6].

IL-28B gene polymorphism currently seems to be one of the strongest predictors of SVR [7].

The aim of this study was to detect IL-28B gene polymorphism in chronic hepatitis C patients in Latvia and to analyze therapy results in patients with different IL-28B gene polymorphisms. This is the first study carried out in Latvia, in the Baltic States and likely in Eastern Europe for the detection of Interleukin 28B gene polymorphism and how it impacts on hepatitis C treatment.

## 2. Material and Methods

There were 159 chronic viral hepatitis C patients included in the study. All chronic viral hepatitis C patients who came to Infectology Center of Latvia for a hepatologist's consultation were tested. Patients had to sign the informed patients' consent prior to testing. In order to detect IL-28B gene polymorphism—rs12979860 genotype frequency we used molecular biology methods: classical DNA separation from blood samples by phenol, amplification by PCR, and standard sequencing by Big Dye (Applied Biosystems). Genotype was defined as CC, CT, TC, or TT type.

142 patients were treated with the standard of care therapy-pegylated interferon in combination with ribavirin. 80 percent of patients received pegylated interferon *α*2a and 20 percent—*α*2b. We analyzed therapy results according to IL-28B gene polymorphism. All patients were divided into 3 subgroups: CC, CT/TC, and TT. Different patient factors, laboratory tests, viral kinetics, and treatment response were analyzed. In terms of therapy result patients were divided into 3 groups: responders, nonresponders, and interrupters. Responders achieved sustained virological response and were HCV-RNA negative at the end of therapy and 24 weeks after stopping treatment. Nonresponders were HCV-RNA positive at week 12 (null responders), at the end of therapy (partial responders) or at week 24 after stopping therapy (relapsers). Interrupters stopped therapy due to financial reason (there is 75% drug reimbursement for chronic hepatitis C treatment, 25% of treatment expenses are covered by patient) or due to unknown reason. HCV-RNA was measured using polymerase chain reaction (PCR), AMPLICOR, version 2.0; Roche, USA. Viral load for genotype 1 patients was measured at baseline and at week 12 on treatment. Liver biopsy was performed for 126 (88.7%) patients, and morphological assessment was done using *Knodell's* HAI index. There were not statistically significant differences between groups with regard to fibrosis stage and HAI index.

Statistical processing of data was done using SPSS v.15.0 and Microsoft Office Excel v.11. Comparison between groups was performed using *Pearson Chi-Square* test or *Fisher's Exact* test. Significance was defined at a *P* value of less than 0.05.

Study was approved by the Independent Ethics Committee for clinical investigation of drugs and pharmaceutical products in Latvia.

Study was performed during the 2009–2011 at the State Agency “Infectology center of Latvia.”

## 3. Results and Discussion

**Figure 1:**
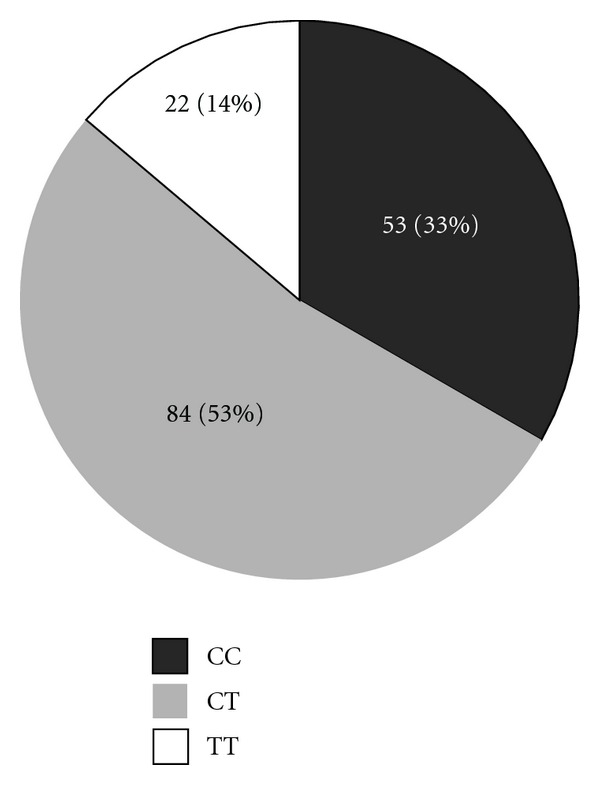
Distribution of IL-28B gene polymorphism.

There were 53 patients (33%) with CC genotype, 84 patients (53%) with CT (83 patients) or TC (1 patient) genotype, and 22 patients (14%) with TT genotype among chronic viral hepatitis C patients in Latvia (Figure 1).

142 patients were treated with the standard of care therapy-pegylated interferon in combination with ribavirin Table 1.

All included patients were Caucasians, a majority of them male (59%) and genotype 1 (61%) patients.

**Figure 2:**
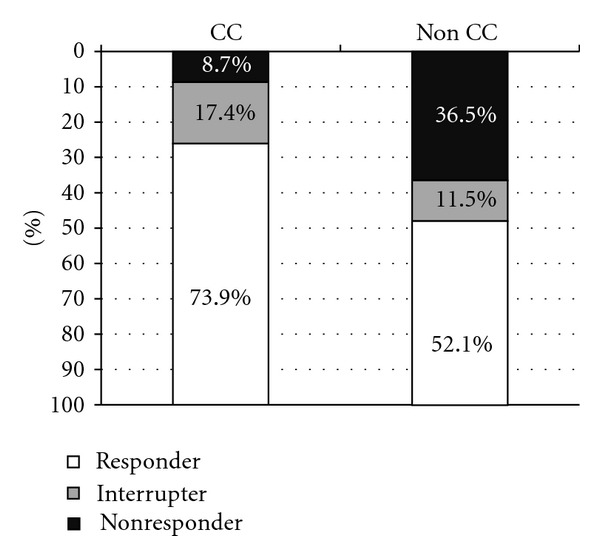
Treatment results depending on IL-28B gene polymorphism.

34 patients (74%) in CC genotype subgroup achieved SVR versus 50 patients (52%) in non-CC subgroup—41 patient in CT/TC and 9 patients in TT subgroups. There were 4 patients (8.7%) nonresponders in CC subgroup (all were relapsers: 3 of them with HCV genotype 1, 1 patient—HCV genotype 2) versus 35 patients (36.5%) in non-CC subgroups (7 patients null responders, HCV genotype 1, 13 patients partial responders: 11 patients HCV genotype 1, 2 patients HCV genotype 3, and 15 patients relapsers, HCV genotype 1); the differences were statistically significant, *P* = 0.002*, Pearson Chi-Square* test (Figure 2). 

There was statistically significant difference also in patients with genotype 1 HCV infection—SVR which was achieved in 16 patients (16/19, 84%) in CC subgroup versus 30 patients (30/63, 47.6%) in non-CC subgroups, *P* = 0.007*, Fisher's Exact* test (Table 2).

The last two decades have seen major advances in HCV treatment options and patient management. However, the standard of care treatment (peg interferon plus ribavirin) is effective in less than half of chronically infected HCV genotype 1 treatment naïve patients, regardless of the type or dose of peg interferon used [8–10].

There are many factors influencing therapy result; sometimes it is hard to understand which of them is the most essential. During recent years there are publications of interleukin 28B gene polymorphism as a strong predictor of SVR in patients treated with pegylated interferon and ribavirin.

According to Thompson et al. data [7], published in 2010, IL-28B gene polymorphism between Caucasians is distributed as follows: CC 37%, CT 51%, and TT 12%. In Latvia majority of people are Caucasians. These results are similar to our data: CC33%, CT 53%, and TT 14%. SVR rates achieved in Thompson's study are 69% of patients in CC group, 33% in CT group, and 27% in TT group [7]. In our study these data are different: 74%, 55%, and 43%, respectively. This difference is probably because of different patient profile: we included all genotype patients (61% were 1st genotype), but in Thompson's study there were only 1st genotype patients included, and it is already known that SVR rates are better in patients with 2nd and 3rd genotypes.

Par et al. [11] have published data in CEMED where CC genotype in HCV patients was noted with significantly lower frequency than in healthy controls (79/281, 28.11% versus 54/104, 51.92%), suggesting a protective role of this variant. Also patients treated with pegylated interferon and ribavirin with CC genotype achieved SVR at significantly higher rates, than those with TT genotype (25/46, 54.34% versus 7/24, 29.16%).

If we analyze only HCV genotype 1 patients, our data shows slightly better results in comparison with Thompson and Par studies. In CC subgroup SVR was achieved in 69% (Thompson), 54.34% (Par), and 84% in our study.

In addition, in our study we analyzed other different treatment efficacy factors, including number of missed treatment doses. 80 percent of patients did not miss any dose. We have to stress that chronic viral hepatitis C patients are paying 25% of treatment costs themselves in Latvia, and 75% is covered by sick fund. Actually they are highly motivated to use assigned medicine.

In terms of the higher SVR on CC genotype patients from the study cohort when compared with Thompson et al.' 2010 study, this cohort had younger patients with lower BMI's, but with higher advanced fibrosis and steatosis rate. Besides that, fibrosis stage was higher in nonresponders group, in comparison to responders group, *P* = 0.029*, Mann-Whitney U-test*. These factors influence SVR rates in hepatitis C patients and could have played a role as well in the difference from virologic responses.

Our study is the first study in Latvia and Baltics done on interleukin B28 gene polymorphism with findings similar to other studies involving Caucasian patients.

## 4. Conclusions

While the most prevalent genotype of IL-28B in Latvia is CT, the most favorable response to treatment and SVR is seen in genotype CC patients. IL-28B gene polymorphism is therefore a strong predictor of response to therapy.

## Figures and Tables

**Table 1:** Baseline characteristics of the patient's profile.

	CC	NON-CC	TOTAL
Average age, years	35	37	37 (18–68)
No. of patients, >40years	15 (33%)	40 (42%)	54 (39%)
Male sex	27 (59%)	57 (59%)	84 (59%)
BMI, kg/m^2^	25.2	26.2	25.9
BMI, >30 kg/m^2^	5 (11%)	12 (13%)	17 (12%)
Genotype 1	21 (46%)	66 (69%)	87 (61%)
Genotype 2, 3	25 (54%)	30 (31%)	55 (39%)
HCV-RNA, ×10^6^ IU/mL (Genotype1)	2.78	2.19	2.33
HCV-RNA, >600,000 IU/mL (Genotype 1)	18 (39%)	47 (49%)	65 (46%)
ALT, U/L	112 (17–325)	104 (22–447)	106 (17–447)
ALT, >ULN	43 (93%)	85 (88%)	128 (90%)
GGT, U/L	46.8 (9–228)	88 (6–526)	75 (6–526)
GGT, >ULN	11 (24%)	40 (42%)	51 (36%)
Cholesterol, mM/L	4.14 (2.09–7.35)	4.6 (2.46–8.17)	4.48 (2.09–8.17)
Triglycerides, mM/L	1.17 (0.28–4.06)	1.17 (0.3–5.44)	1.11 (0.28–5.44)
Liver fibrosis* (*Knodell*) F0 F1 F3 F4	0.975 (0–3) 9 (21.9%) 28 (68.3%) 4 (9.7%) 0	1.2 (0–4) 21 (24.7%) 47 (55.3%) 12 (14%) 5 (5.9%)	1.13 (0–4) 30 (23.8%) 75 (59.5%) 16 (12.7%) 5 (3.9%)
HAI index* (*Knodell*)	6.44 (1–12)	6.54 (2–13)	6.5 (1–13)
Steatosis* > grade 0	34 (83%)	65 (76.5%)	99 (78.6%)
SVR	34 (74%)	50 (52%)	84 (59%)

*Missing data: histology *n* = 11 (non-CC), *n* = 5 (CC).

**Table 2:** Treatment results in patients with genotype 1 depending on IL-28B gene polymorphism.

			IL-28B genotype		Total	*P*
			CC	Non-CC		
Therapy result Genotype 1	Nonresponders	Count % within IL-28B genotype	3 15,79%	33 52,38%	36 43,90%	
	Responders	Count % within IL-28B genotype	16 84,21%	30 47,62%	46 56,10%	0.007367

Total		Count % within IL-28B genotype	19 100%	63 100%	82 100%	
